# Prenatal Detection of Interatrial Septal Aneurysm With Postnatal Follow-Up: A Rare Pediatric Case of Arrhythmia in a Two-Year-Old Male

**DOI:** 10.7759/cureus.106728

**Published:** 2026-04-09

**Authors:** Attaa K Taha, Mohammed Khayyal, Sahar S Sheta, Mahmoud A Ismaiel

**Affiliations:** 1 Echo Imaging, University of Baghdad College of Medicine, Baghdad, IRQ; 2 Cardiology, Mansoura Insurance Hospital, Mansoura, EGY; 3 Cardiology, Armed Forces Hospital - Southern Region, Khamis Mushait, SAU; 4 Pediatric Cardiology, Cairo University, Giza, EGY; 5 Cardiovascular, Royal Free Hospital, London, GBR

**Keywords:** atrial septal aneurysm, fetal arrhythmia, fetal echocardiography, pediatric echocardiography, supraventricular arrhythmia

## Abstract

Interatrial septal aneurysm (IASA) is not uncommon in fetal and neonatal screening echocardiography. However, its persistence into childhood is rare. The presence of an IASA was associated with increased risk of arrhythmias and embolic events. Fetal tachycardia was suspected at 34 weeks of gestational age. Fetal echocardiography showed a large IASA with a normal fetal heart rate and no other congenital abnormalities. The case presented again at the age of two years with recurrent syncopal attacks, with confirmed atrioventricular (AV) nodal re-entry tachycardia correlated with the symptoms. Echocardiography showed a large type R1 IASA. The patient was referred to cardiac electrophysiology for further management. Persistence of interatrial aneurysms into childhood is rare. It can be associated with arrhythmogenic complications. A new class of classification of IASA dedicated to fetal diagnosis is suggested.

## Introduction

An interatrial septal aneurysm (IASA) is a known congenital or acquired abnormality characterized by a localized, saccular protrusion of the interatrial septum (IAS) into one or both atrial chambers. It was first described by Lang and Posselt in 1934 as a postmortem finding associated with redundancy and thinning of the fossa ovalis region of the septum [1]. The prevalence of IASA in the neonates is estimated at 7.6% in a screening study conducted by Ozcelik et al. in 2006 [2]. Anatomically, the aneurysm usually arises from the region of the fossa ovalis, representing excessive mobility or tissue redundancy of the septum primum. It can occur in isolation or in association with interatrial communications, such as patent foramen ovale (PFO) or atrial septal defect (ASD). Histologically, the aneurysmal tissue shows attenuation of muscular fibers and increased connective tissue, predisposing to abnormal motion and potential arrhythmogenicity [3,4].

Olivares-Reyes et al. proposed the most widely accepted echocardiographic classification of IASA based on the direction and extent of septal excursion seen in the apical four-chamber view [5]. This classification aids in differentiating IASA subtypes, assessing hemodynamic impact, and correlating morphology with clinical manifestations such as arrhythmia or paradoxical embolism [6].

While IASA is commonly identified in adults undergoing echocardiography for stroke or arrhythmia evaluation, prenatal and pediatric detection remains rare. The availability of advanced fetal echocardiography, guided by the International Society of Ultrasound in Obstetrics and Gynecology (ISUOG) standards, allows early recognition of subtle septal abnormalities and rhythm disturbances in utero. However, the clinical relevance of IASA detected prenatally is still uncertain, particularly regarding its postnatal arrhythmogenic potential [7].

In this report, we present a pediatric case of isolated IASA first detected prenatally and later associated with paroxysmal supraventricular arrhythmias and syncope at the age of two years. This case underscores the value of prenatal echocardiographic detection and emphasizes the importance of longitudinal postnatal rhythm surveillance.

## Case presentation

A 28-year-old, gravida 2, para 1, woman was referred to our fetal cardiology clinic at 34 weeks of gestation after her obstetrician noted fetal tachycardia exceeding 180 beats per minute during routine antenatal evaluation. Upon fetal echocardiographic assessment in our clinic, the heart rate was found to be within normal range (155 bpm), and the rhythm was regular. Therefore, the patient was followed closely with serial assessments.

Fetal Assessment

**Figure 1:**
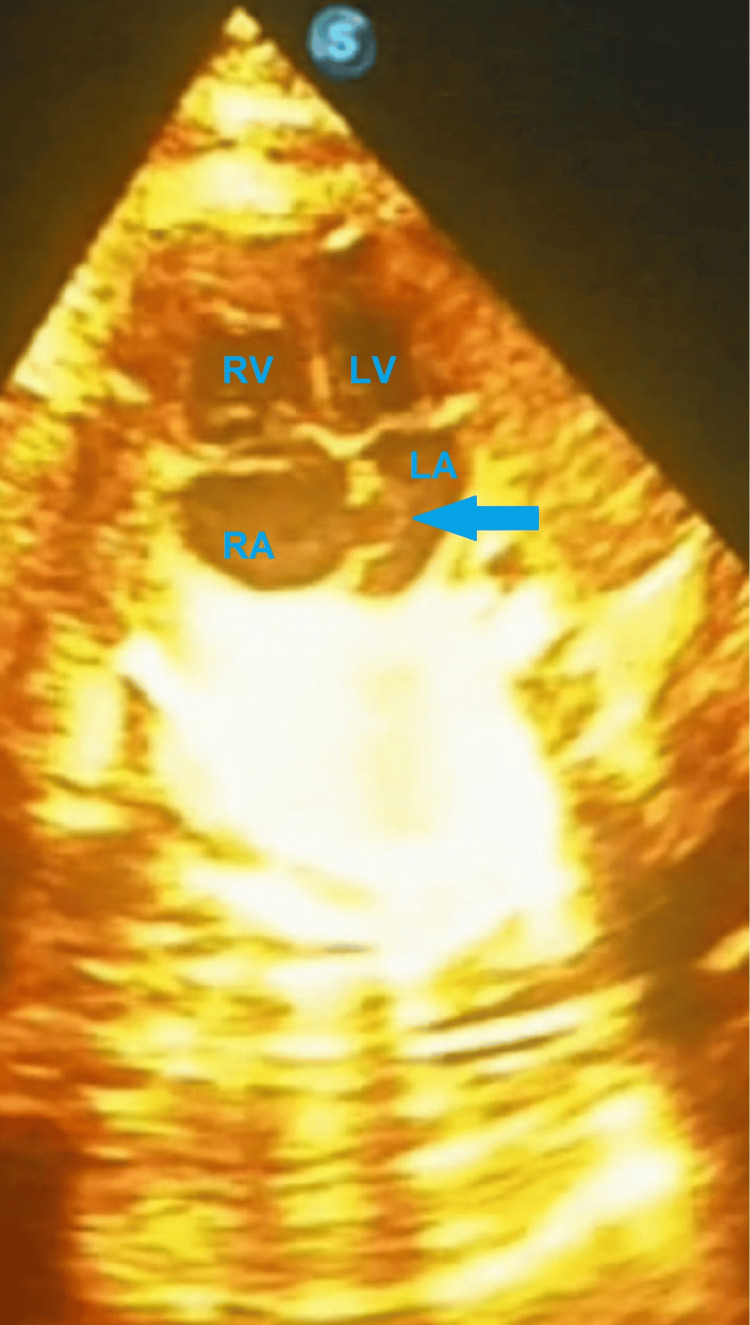
Fetal echocardiographic four-chamber view showing the interatrial septal aneurysm LA: left atrium, LV: left ventricle, RA: right atrium, RV: right ventricle, Arrow: interatrial septum aneurysm

**Figure 2:**
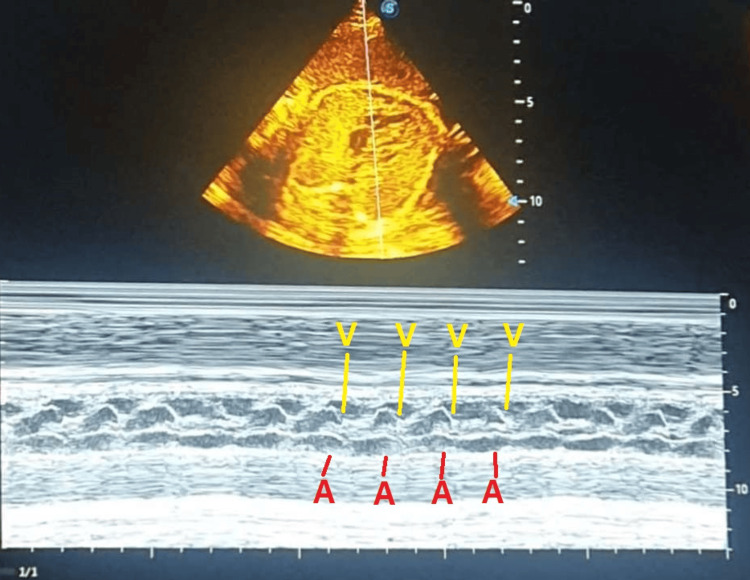
M-mode tracing demonstrating 1:1 atrioventricular conduction A: atrial contraction, V: ventricular contraction

Fetal growth corresponded to gestational age. Fetal echocardiogram revealed an IASA with patent foramen ovale (Figure 1), mild tricuspid insufficiency, and normal cardiac contractility. Heart rate was 155 bpm, with an atrial/ventricular contraction ratio of 1:1, confirmed by M-mode echocardiography (Figure 2). No other structural abnormalities were observed.

Postnatal Period

The child was not evaluated immediately after birth, and no clinical issues were reported during infancy.

**Figure 3:**
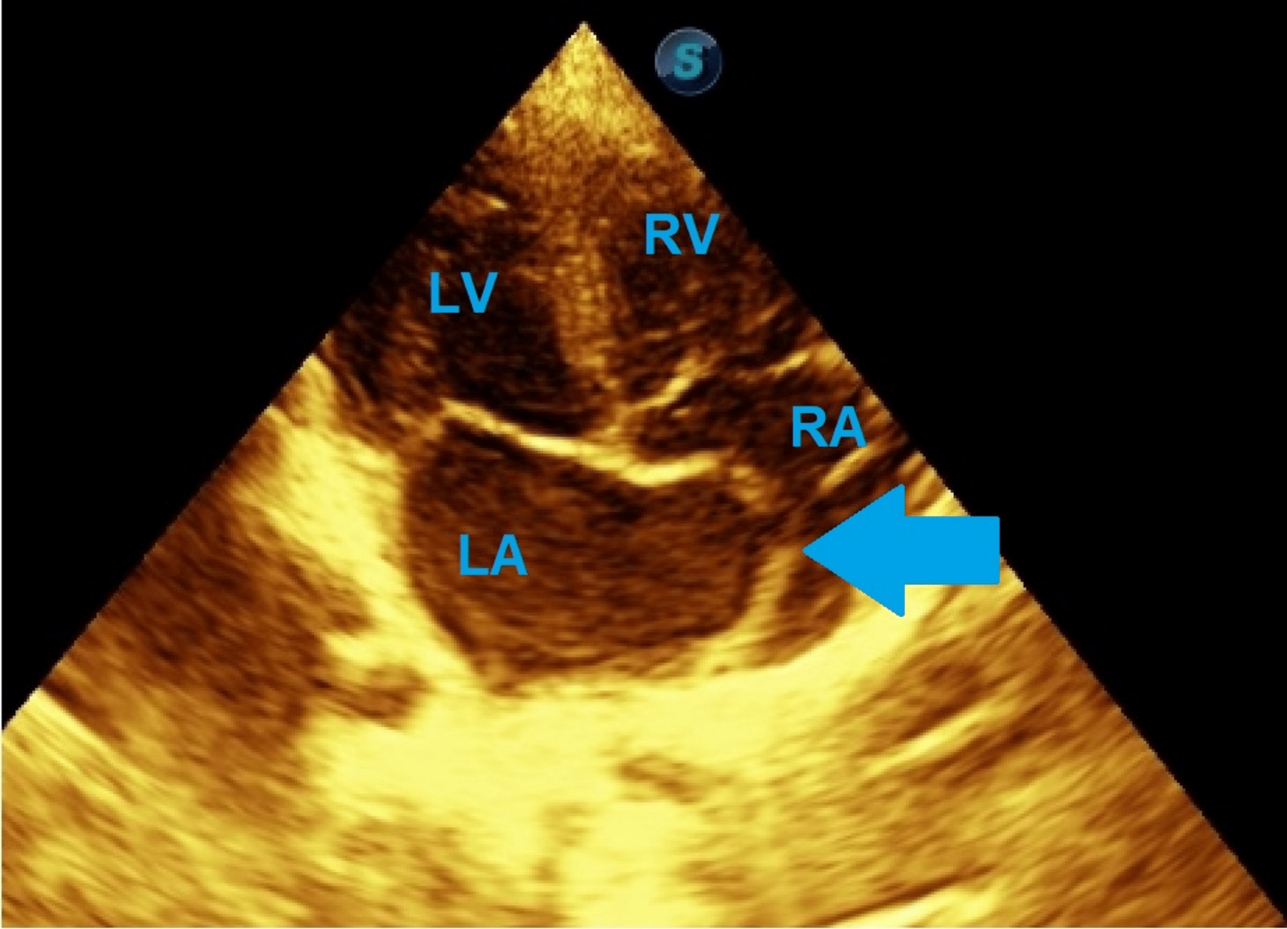
Postnatal transthoracic echocardiogram showing a large mobile aneurysm protruding into the right atrium LA: left atrium, LV: left ventricle, RA: right atrium, RV: right ventricle, Arrow: interatrial septum aneurysm

**Figure 4:**
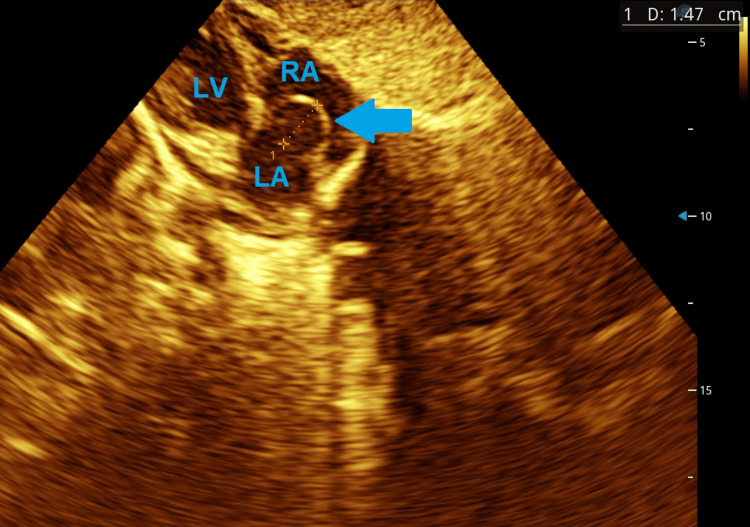
Postnatal subcostal view showing a large mobile aneurysm protruding into the right atrium LA: left atrium, LV: left ventricle, RA: right atrium, Arrow: interatrial septum aneurysm

At age two years, the child developed recurrent, brief syncopal episodes. Physical and neurological examinations were unremarkable. Baseline electrocardiogram demonstrated sinus rhythm. Twenty-four-hour Holter monitoring revealed AV nodal re-entry tachycardia correlating with symptomatic episodes. Echocardiography showed a large, mobile IASA protruding into the right atrium (Figures 3-4), with normal ventricular function and valves (Videos 1-4). The IASA is type 1R. Table 1 presents the Olivares-Reyes echocardiographic classification of IASA [5].

**Video 1:** Apical view showing a large interatrial septal aneurysm in the postnatal echo study

**Video 2:** Subcostal view showing a large interatrial septal aneurysm in the postnatal echo study

**Video 3:** Modified bicaval subcostal view showing a large interatrial septal aneurysm in the postnatal echo study

**Video 4:** Postnatal echo with color Doppler showing no flow through the atrial septal aneurysm with mild tricuspid regurgitation

**Table 1:** Olivares-Reyes echocardiographic classification of interatrial septal aneurysm Source: [5]

Type	Description
Type 1R	Aneurysm protrudes exclusively into the right atrium.
Type 2L	Aneurysm protrudes exclusively into the left atrium.
Type 3RL	Bidirectional excursion, predominantly toward the right atrium.
Type 4LR	Bidirectional excursion, predominantly toward the left atrium.
Type 5	Aneurysm moves equally in both directions.

The child was referred to a cardiac electrophysiology-specialized center for further assessment and management. The parents were counseled regarding structured long-term follow-up.

## Discussion

The IAS develops through a complex process during fetal life. This process involves the fusion of the septum primum and the septum secondum, leaving a valve-like opening between them known as the foramen ovale. The presence of the foramen ovale is essential for fetal circulation and hemodynamics, to allow the passage of blood with high oxygen content from the right atrium to the left atrium directed by higher pressure in the right atrium [8]. After delivery and expansion of the lungs in neonatal life, the pressure in right-sided chambers drops below the pressure in left-sided chambers. This causes functional closure of the foramen ovale [9].

IASA represents a morphologic abnormality of the atrial septum characterized by excessive mobility or localized bulging of the fossa ovalis region, which may predispose to arrhythmias and embolic events [5]. Although commonly recognized in adults during evaluation for stroke or atrial arrhythmias, its prenatal detection remains rare, and the postnatal course is not well defined [7].

In the present case, the referral was for evaluation after fetal tachycardia exceeding 180 bpm was noted by the obstetrician. Upon examination, the heart rate normalized, yet the finding of an IASA prompted longitudinal follow-up, underscoring the importance of fetal echocardiography not only in structural assessment but also in rhythm evaluation.

Prenatal IASA may be transient, often associated with redundancy of the septum primum and hemodynamic forces between the right and left atria in late gestation [2]. Most of the neonatal and fetal cases of IASA regressed spontaneously postnatally without clinical sequelae [10]. However, as in our patient, persistent and mobile aneurysms may serve as a substrate for arrhythmogenesis later in life.

The pathophysiology of arrhythmia in IASA is multifactorial. The aneurysmal motion may cause mechanical stretch and distortion of atrial tissue, leading to altered conduction properties, micro-reentry circuits, or ectopic automatic foci along the aneurysmal rim. Additionally, the redundant septal tissue may interfere with the normal interatrial conduction pathways, particularly the Bachmann bundle, predisposing to supraventricular tachycardia or atrial flutter [11]. This is supported by Holter findings in our patient, showing paroxysmal atrial tachyarrhythmias that correlated temporally with syncopal episodes.

The Olivares-Reyes classification, applied in postnatal echocardiography, is useful to describe the direction of septal excursion [5]. Our patient likely corresponded to type 1R, characterized by motion toward the right atrium. It is important to note that the Olivares-Reyes classification was originally developed for adult echocardiographic evaluation, reflecting postnatal hemodynamic conditions [5]. In the fetus, where right atrial dominance and foramen ovale shunting are physiologic, septal motion and excursion patterns differ significantly. Therefore, this classification may not be directly applicable prenatally, and a modified "type 0" could conceptually describe the distinctive fetal hemodynamic pattern until postnatal adaptation occurs. The proposed modification to the Olivares-Reyes classification takes into consideration the different hemodynamic and physiologic conditions during fetal life.

Several reports have described the association between IASA and atrial arrhythmias in pediatric patients [11-15]. Deveci et al. documented that up to 43.9% of adults with IASA presented with supraventricular tachycardia, typically benign and self-limited [12]. Our findings are consistent with this observation, as arrhythmias were transient and did not require pharmacologic intervention. Nonetheless, the presence of syncope in a young child warranted careful rhythm evaluation and cardiology follow-up.

From a developmental standpoint, fetal IASA may reflect physiologic hemodynamic dominance of the right atrium in late gestation, where the foramen ovale shunts oxygenated blood toward the left atrium. After birth, changes in atrial pressures normally flatten the septum primum; persistence of aneurysmal motion suggests structural redundancy rather than transient hemodynamic imbalance. This may explain why the same structural abnormality later becomes a potential arrhythmogenic focus after birth [16].

The ISUOG guidelines emphasize the value of systematic fetal cardiac screening, including rhythm and septal motion assessment [7]. This case supports those recommendations: an initially benign prenatal finding became clinically significant postnatally. Such observations highlight the continuum between prenatal echocardiographic morphology and postnatal electrophysiologic behavior, justifying periodic rhythm monitoring even in asymptomatic infants.

Our management strategy was conservative, as recommended in the literature. In the absence of thromboembolic risk or significant shunt, anticoagulation and surgical intervention are not indicated. Antiarrhythmic therapy is reserved for sustained or symptomatic arrhythmias. Continuous follow-up allows early recognition of changes in rhythm or septal mobility.

Ultimately, this case illustrates that IASA, though often considered benign, may evolve dynamically over time - from a silent prenatal finding to a clinically relevant arrhythmic substrate in early childhood. Awareness of this potential progression and early incorporation of fetal echocardiography findings into postnatal care plans can improve outcomes through anticipatory surveillance and parental counseling.

## Conclusions

Prenatal detection of IASA via fetal echocardiography allows early identification of structural anomalies. Fetal echocardiography enables precise assessment of cardiac rhythm, clarifies the underlying mechanisms of arrhythmias, and confidently excludes associated structural abnormalities. Isolated IASA can manifest with arrhythmia and syncope in early childhood, emphasizing the importance of longitudinal postnatal follow-up guided by ISUOG protocols.
